# Supplementary material for: Large-scale genomic rearrangements boost SCRaMbLE in Saccharomyces cerevisiae
Source: Nat Commun. 2024 Jan 26;15:770. doi: 10.1038/s41467-023-44511-5 (PMC10817965; doi:10.1038/s41467-023-44511-5)
Supplement: Supplementary file 1 — Supplementary information [file 41467_2023_44511_MOESM1_ESM.pdf]

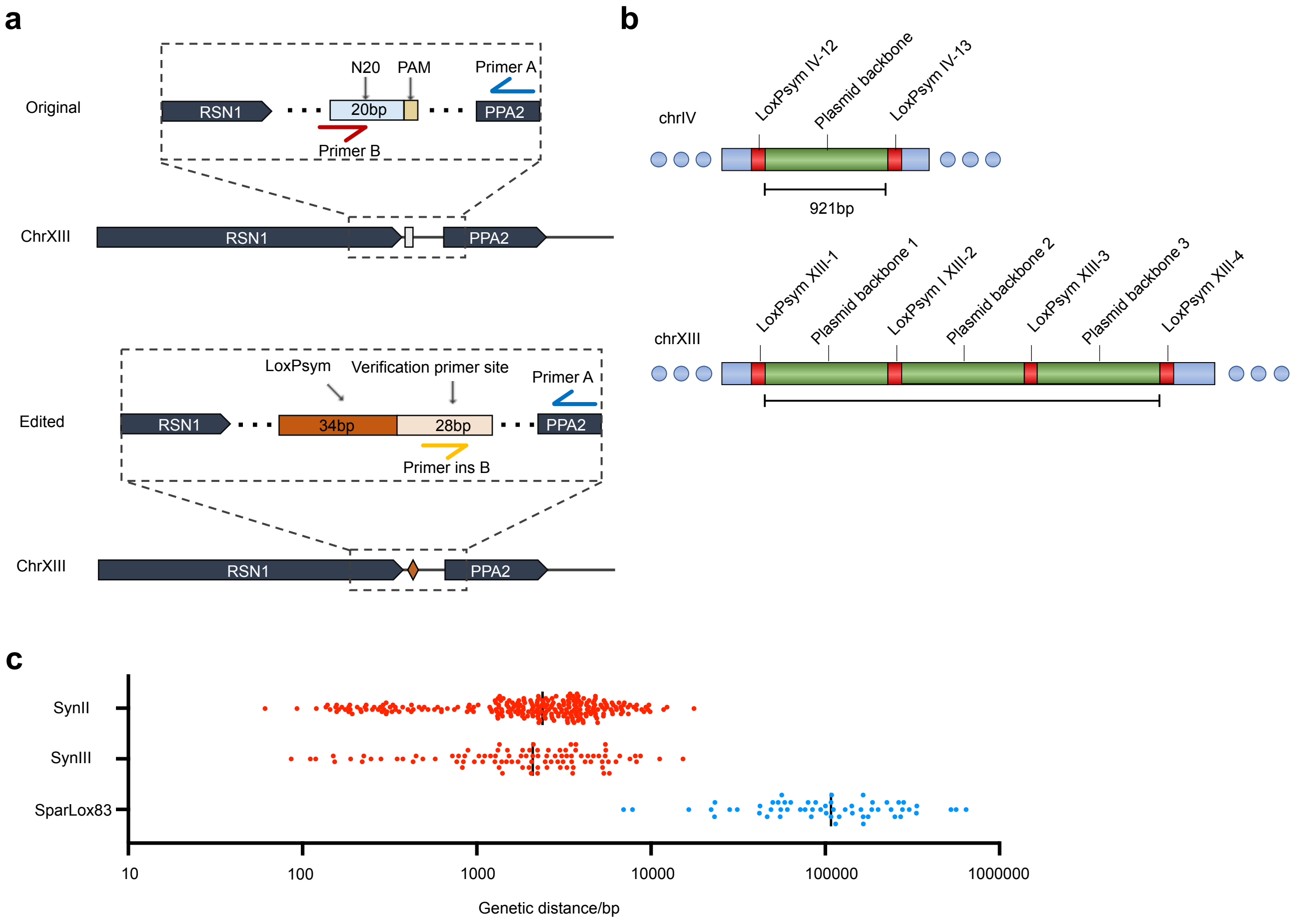

Supplementary Fig. 1 | Selection of loxPsym insertion sites. a. Example of loxPsym insertion site selection. Intergenic regions of the yeast genome were used as inputs to search for possible crRNA (protospacer) sequences for Cas9 nuclease (denoted N20) at http://crispr-era.stanford.edu/. Next, the secondary structure of gRNAs with the highest scores according to http://rna.tbi.univie.ac.at/cgi-bin/RNAfold.cgi were determined, and sequences lacking a hairpin and with lower minimum free energy structures were selected. For each loxPsym insertion event, primer A was designed within the target locus, primer B was designed adjacent to the sequence being targeted by the insertion event, and primer insB was designed specific to the unique sequence inserted with the loxPsym site. When loxPsym sites were inserted correctly (*e.g.*, SparLox83), primer pair A + B would not produce an ampliconfrom genomic DNA, but primer pair A + insB would produce amplicons of the expected sizes. **b.** The detailed information for the multiple sites at the same loci through self-duplication of the plasmid backbone sequence which were indicated as a red rectangle in Fig. 1a. **c.** Genetic distance between two adjacent loxPsym is different between synthetic chromosomes and SparLox83. Each dot represents a pair of adjacent loxPsym sites. Source data are provided as a Source Data file.

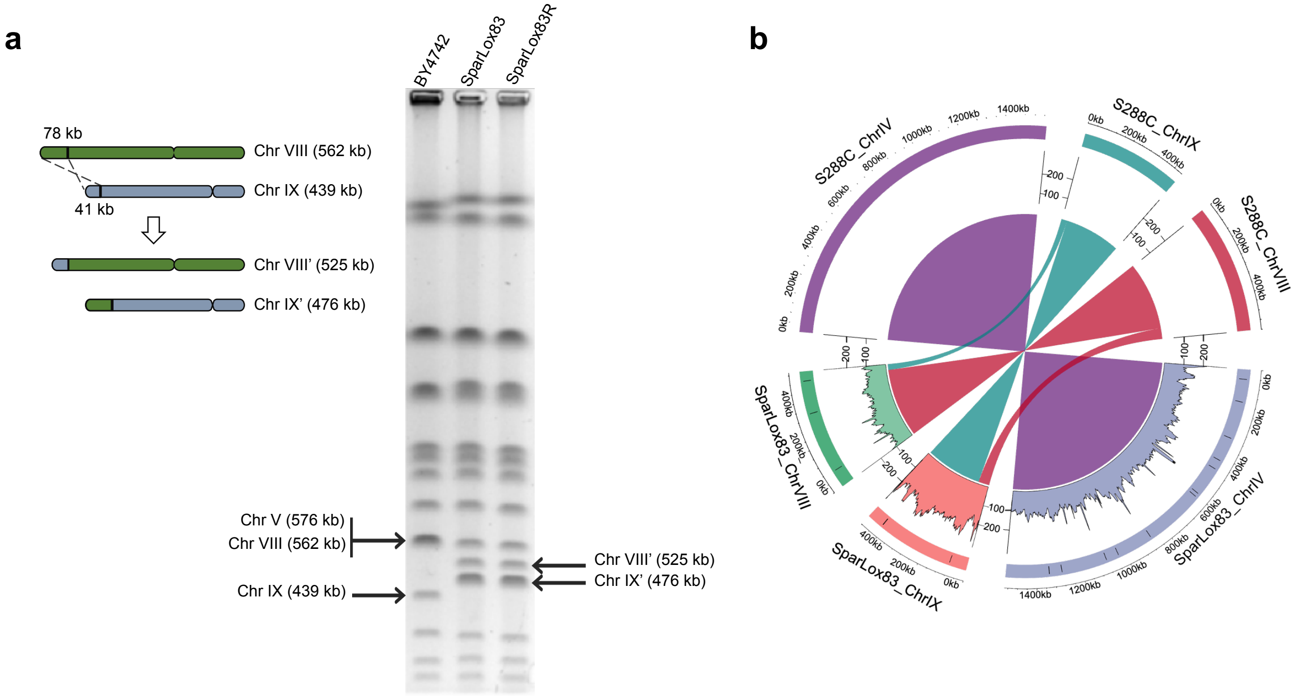

Supplementary Fig. 2 | Translocation occurring during strain construction. a. Pulsed-field gel electrophoresis analysis of a translocation between chromosomes VIII and IX in SparLox83 and SparLox83R. This translocation occurred between the inserted loxPsym sites VIII-3 and IX-1 and resulted in size alterations to both chromosomes (arrows). **b.** Colinearity mapping between chrIV, chrIX and chrVIII in SparLox83 with chrIV (purple), chrIX (green) and chrVIII (red) of the reference genome. The outer circle represents chromosomes before (upper half) and after (lower half) translocation. Black bars in the outer circle indicate the locations of loxPsym sites. The middle circle is a depth plot of the Nanopore sequencing data, and the inner circle indicates the inferred structural variants.

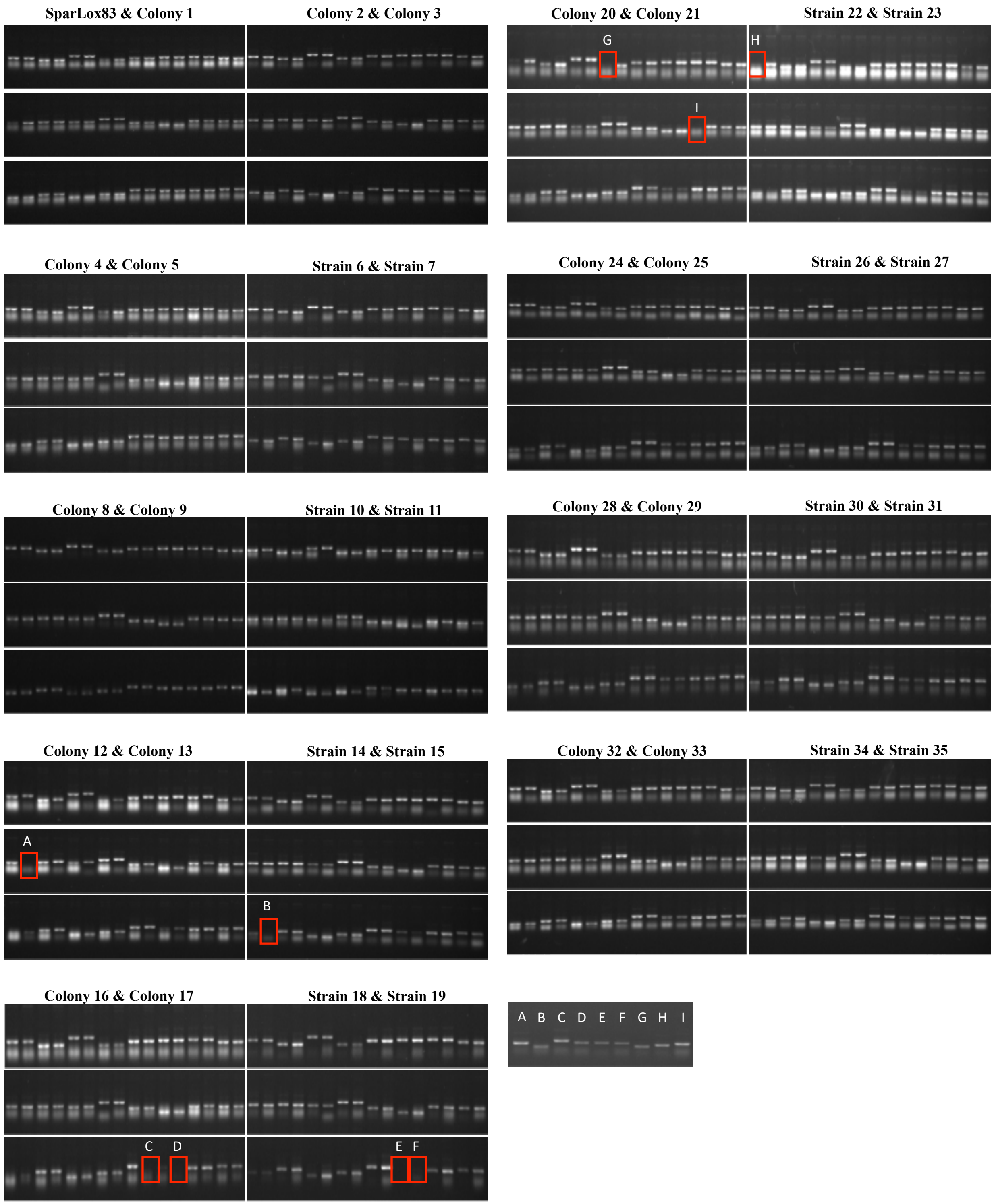

Supplementary Fig. 3 | PCR verification for 24 loxPsym sites in 35 independent colonies after ~125 mitotic generations. The absence of amplicons in the initial amplification was indicated by the red rectangles, while subsequent repetitions revealed the presence of correct amplicons, displayed at the bottom right corner.

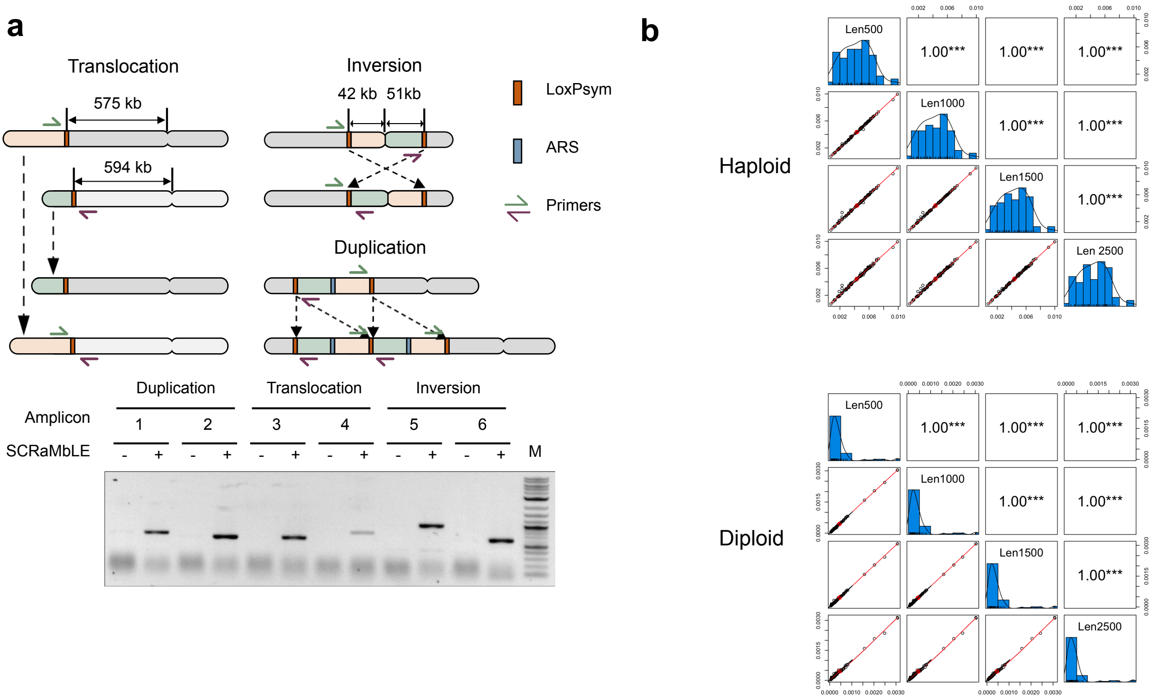

Supplementary Fig. 4 | Junction PCR and Nanopore sequencing revealed diverse rearrangements in SparLox83R after SCRaMbLE. a. Junction PCR to diagnose junctions formed from different types of rearrangement. Examples of the following are shown: translocation, with two loxPsym sites on different chromosomes at similar distances from their original centromeres; inversion, with two loxPsym sites on different arms of the same chromosome at similar distances from the centromere; and duplication, with the fragment between two loxPsym sites containing at least one autonomously replicating sequence (ARS). Primer pairs were designed to generate 500–1000 bp amplicons only upon rearrangement. Amplicon 1, duplication mediated by XIII-5 and XIII-6; amplicon 2, duplication mediated by IV-1 and IV-2; amplicon 3, translocation mediated by IV-9 and XII-6; amplicon 4, translocation mediated by VII-2 and X-4; amplicon 5, inversion mediated by X-5 and X-6; and amplicon 6, inversion mediated by I-2 and I-3. Genomic template DNA was from SparLox83R (-) or the rearrangement induction population (+). Primers are listed in Supplementary Data 4. **b.** ARR calculated using different flank region lengths in haploid and diploid cells. Pearson correlation coefficients within the four lengths in haploid and diploid cells were analyzed. ***p<0.001. Source data are provided as a Source Data file.

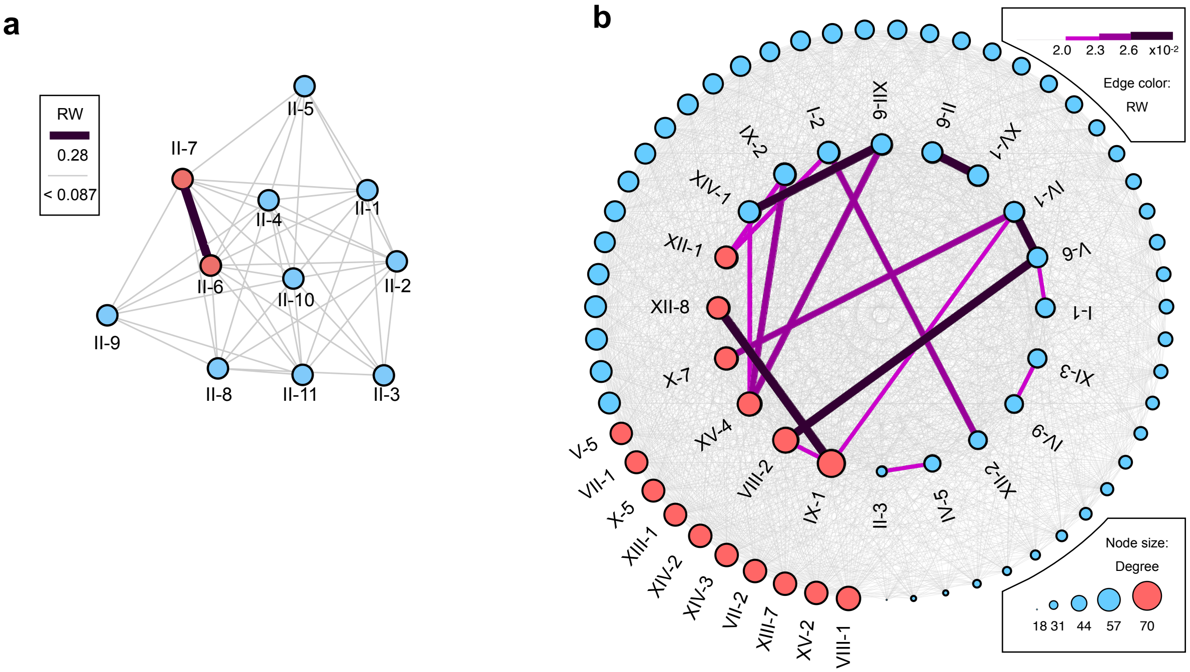

Supplementary Fig. 5 | Uneven distribution of rearrangement frequencies at different loxP sites. a. Intrachromosomal rearrangement network for chrII, representing possible rearrangements among the eleven loxPsym sites (nodes). Pairs of loxPsym sites are shown connected if a rearrangement was detected. Line thickness is proportional to the number of rearrangements detected. The network was generated using Cytoscape (v3.7.1). **b**. Interchromosomal rearrangement network in haploid cells. Node size indicates the number of interconnections with other sites (degree). Red dots indicate degree >= 60. The internal circle consists of dots with rearrangement weights (RW) > 2.0×10^{-2}.

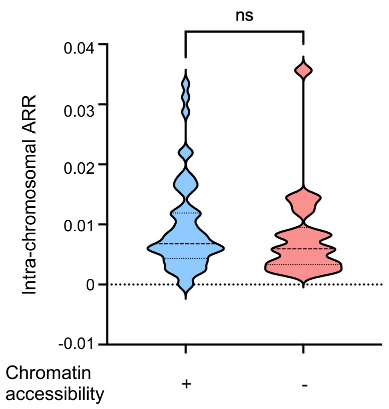

Supplementary Fig. 6 | Comparison of intra-chromosomal ARRs between loxPsym sites within (+) and outside (-) open chromatin regions in haploid cells. Unpaired t-test (two-tail) was used compare the two groups, p=0.4213. Source data are provided as a Source Data file.

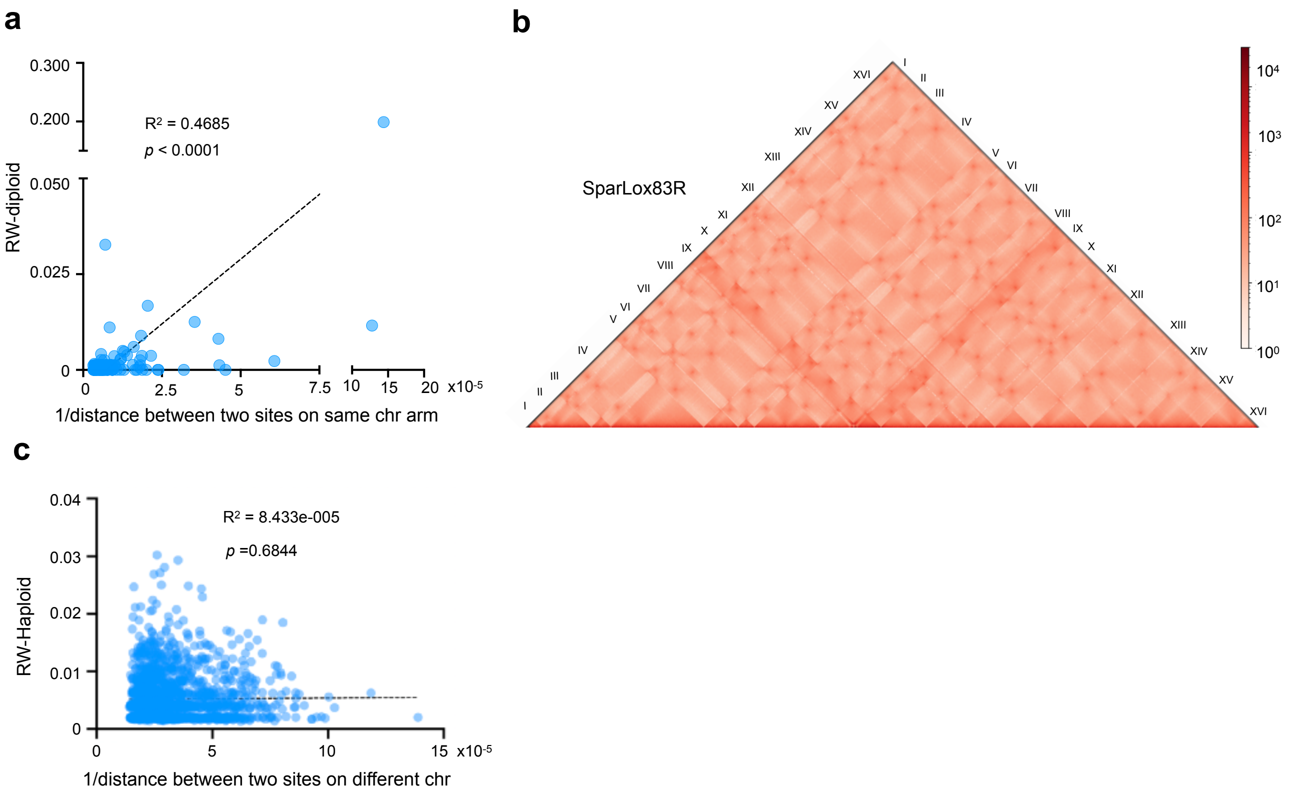

Supplementary Fig. 7 | Correlation between genomic location or 3D spatial distancing of loxPsym sites and their rearrangement event frequencies. a. Correlation between RW in diploid cells and 1/genomic distance between loxPsym sites on the same chromosome arm. Simple linear regression was used. R^2=0.4685 and p<0.0001. Source data are provided as a Source Data file. **b.** KR-normalized Hi-C contact map of SparLox83R. **c.** Correlation between RW in haploid cells and 1/genomic distance between loxPsym sites on the different chromosome. Simple linear regression was used. R^2=8.433e-005 and p=0.6844. Source data are provided as a Source Data file.

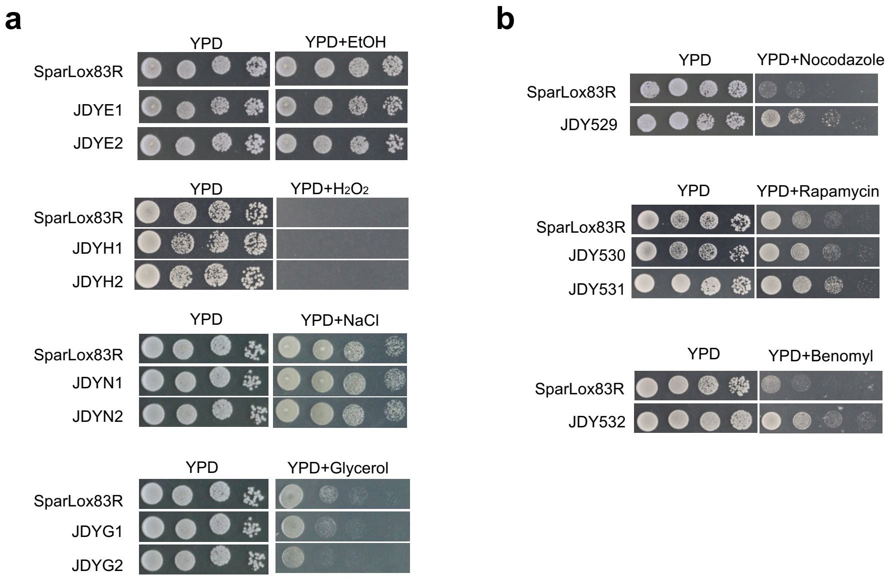

Supplementary Fig. 8 | Serial dilution assay comparing the SCRaMbLEd cells to SparLox83R. a. SCRaMbLEd cells exhibited similar tolerance to the parent strain SparLox83R under stress conditions. Cells at log phase were 10-fold serially diluted onto YPD agar plates containing 10% ethanol, 3 mM H_2O_2, 1 M NaCl, or 3% glycerol and incubated for 2 days at 30°C. **b**. SCRaMbLEd colonies showed more tolerance to nocodazole (10 μg/mL), benomyl (40 μg/mL), or rapamycin (10 ng/mL) than the original strain.

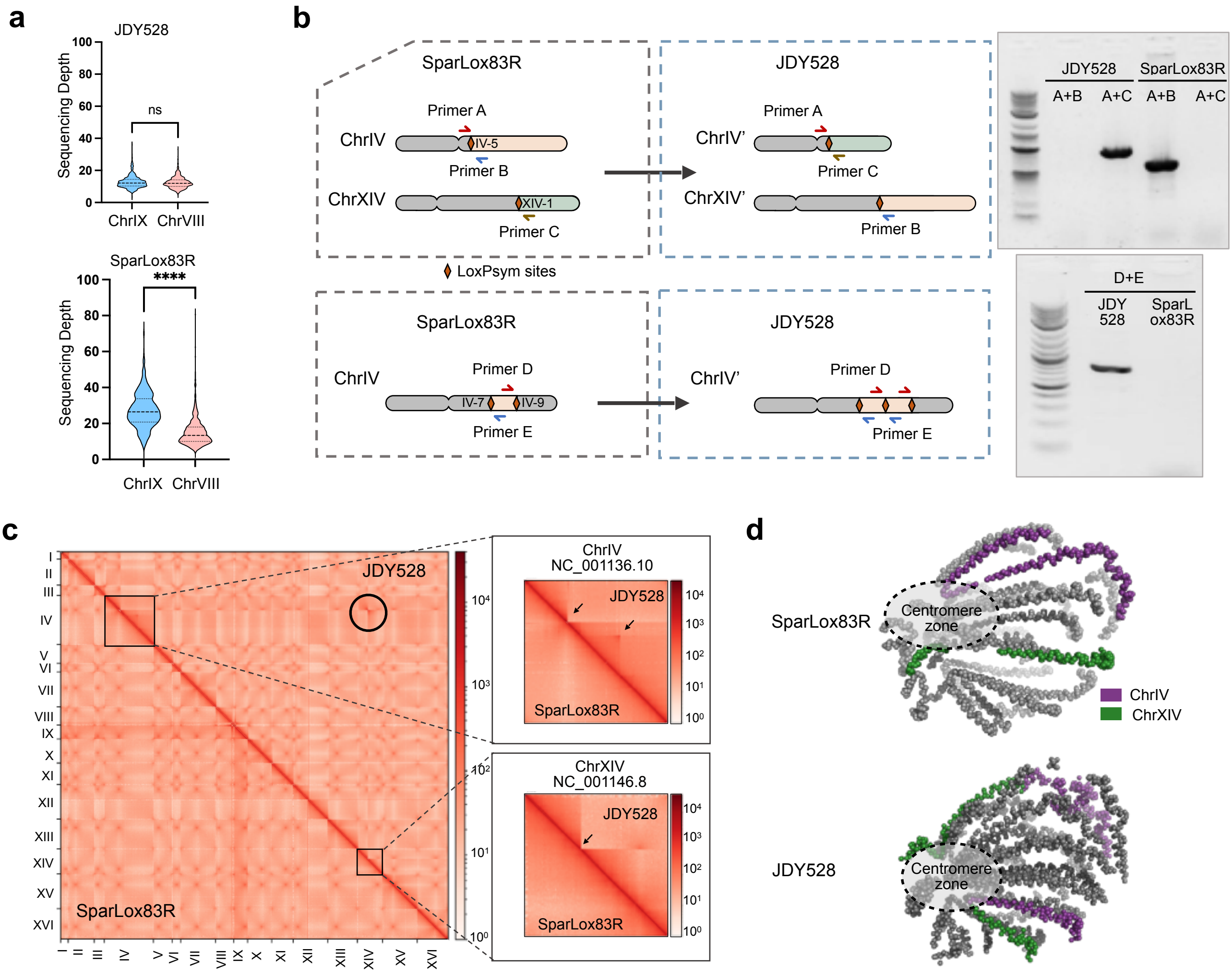

Supplementary Fig. 9 | Exploration of the impact of large-scale rearrangement on phenotype, transcription, and genome 3D structure upon exposure to stress. a. In JDY528, the sequencing depth of chrIX was comparable to that of chrVIII, indicating the copy number variation. Kolmogorov-Smirnov test was used to compare the two groups, p=0.3377 for JDY528 and ****p<0.0001 for SparLox83R. Source data are provided as a Source Data file. **b.** PCR verification of rearrangements in JDY528. Primers are listed in Supplementary Data 4. **c**. Contact maps of the SparLox83R and JDY528 genomes. Translocations and duplications are indicated with black arrows. Red dots in the map indicate centromeres. **d**. Predicted average 3D chromosome localization in SparLox83R and JDY528 based on contact mapping. Rearranged chromosomes are highlighted in different colors.

a

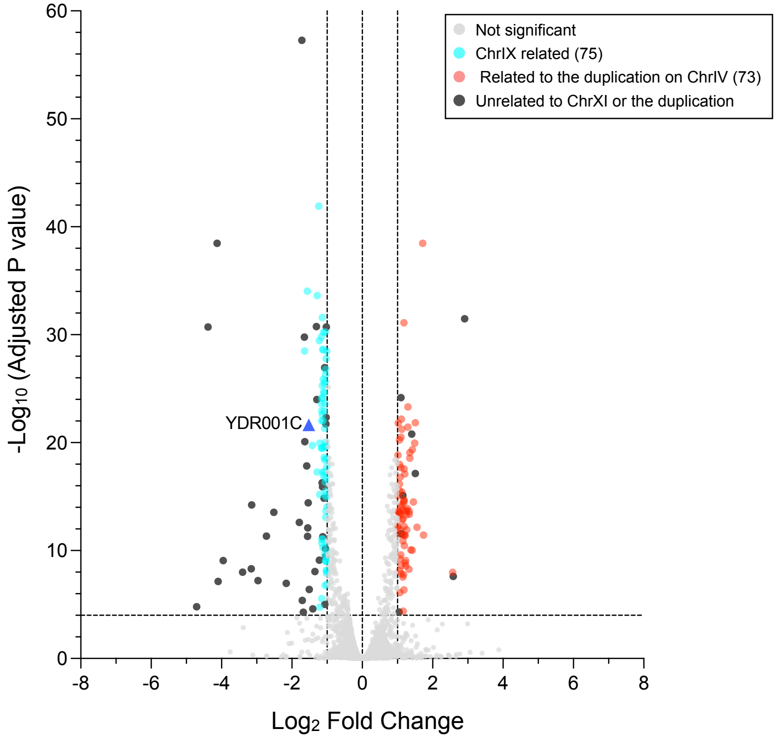

b

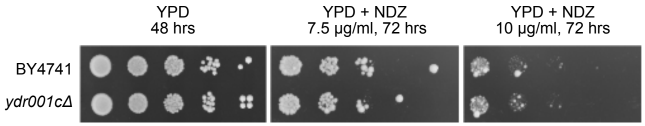

Supplementary Fig. 10 | Transcriptome analysis between JDY528 and SparLox83R. a. Volcano plot of global expression changes of JDY528 compared to SparLox83R. Each dot represents the average of three biological replications. Source data are provided as a Source Data file. Grey dot, genes without change; Green dot, gene located on ChrIX; red dot, genes located in the duplication region; blue triangle, gene related to the rearrangement. **b.** Serial dilution assay comparing the knock-out strain and the wild-type strain.

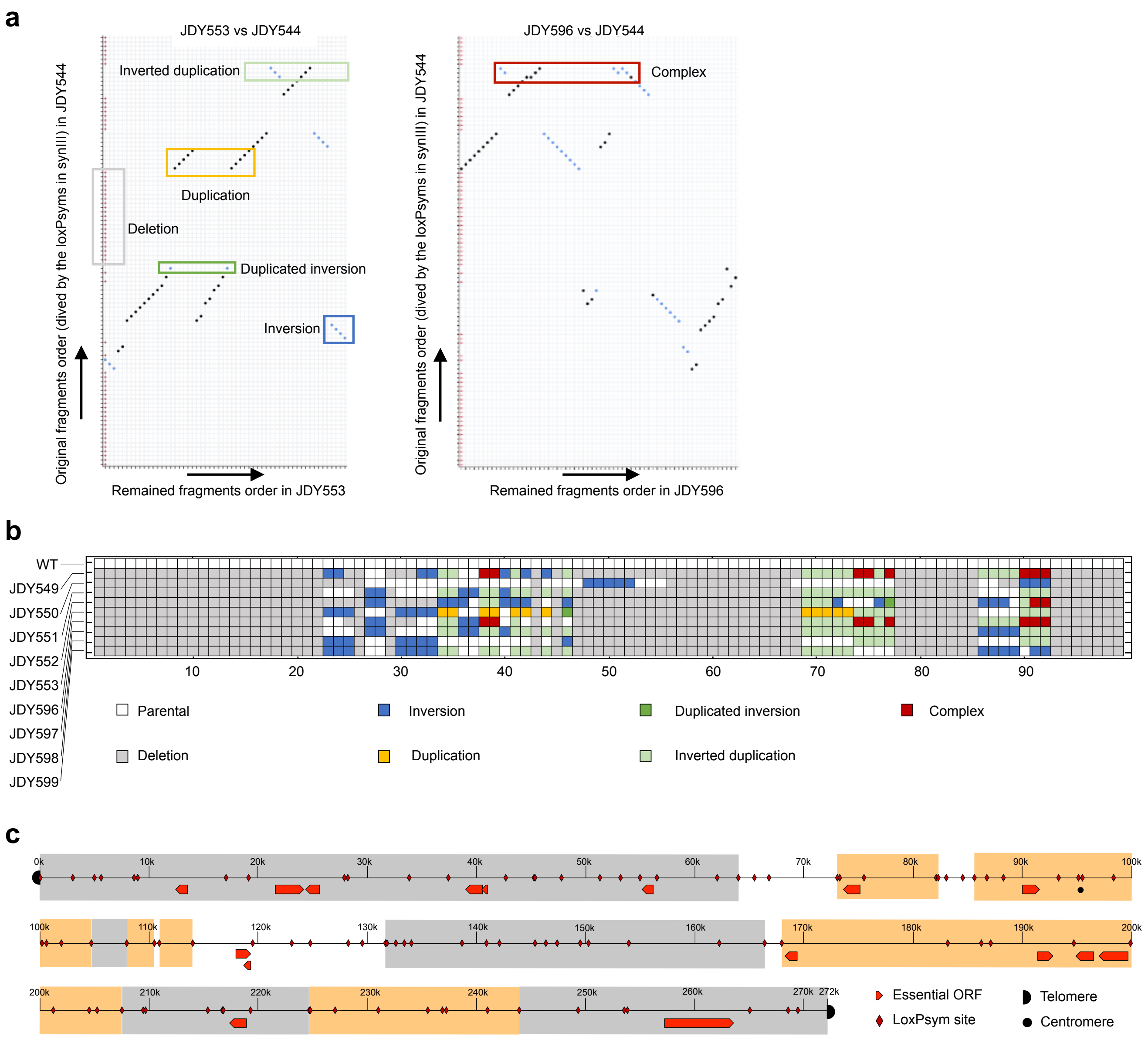

Supplementary Fig. 11 | Rearrangements of synIII were observed in SCRaMbLE cells. a. Dot-plots illustrating synIII rearrangements in JDY553 and JDY596. Each dot represents a fragment between two loxPsym sites. Black dots represent fragments arranged in synIII sequence order and blue dots represent fragments inverted with respect to the synIII sequence. Source data are provided as a Source Data file. **b.** The fate of each segment in each strain was classified as deletion (gray), inversion (blue), duplication (yellow), duplicated inversion (dark green), inverted duplication (light green), multiple duplication (red) or identical to the parent strain (white). **c**. Schematic representation of synIII illustrating common rearrangements among SCRaMbLEants. Regions highlighted in orange represent sequences retained after SCRaMbLE in all SCRaMbLEants. Regions highlighted in gray were lost from synIII in all SCRaMbLEants. Red arrows indicate open reading frames (ORFs) of essential genes.

a

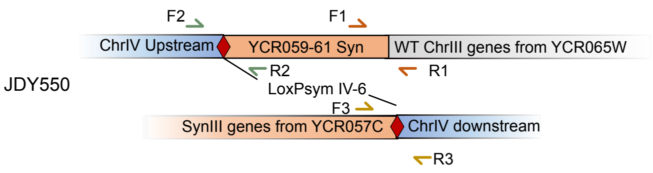

b

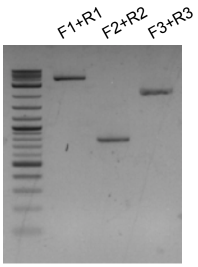

Supplementary Fig. 12 | PCR validation of the rearrangement between synIII and chrIII. a. PCR primers designed to verify translocation between SparLox83R and synIII chromosomes in strain JDY550. **b.** PCR analysis of JDY550. Amplicons were sequenced and the data are shown in Fig 4c. Primers are listed in Supplementary Data 4.

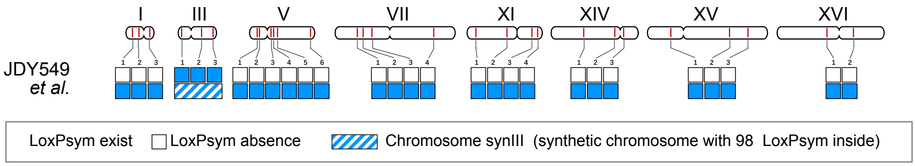

Supplementary Fig. 13 | Copy numbers of 2 kb upstream/downstream regions flanking the loxPsym sequence were determined for chromosomes without rearrangements in the nine diploid strains. The panels exhibited identical in all the nine strains but with varied synIII.

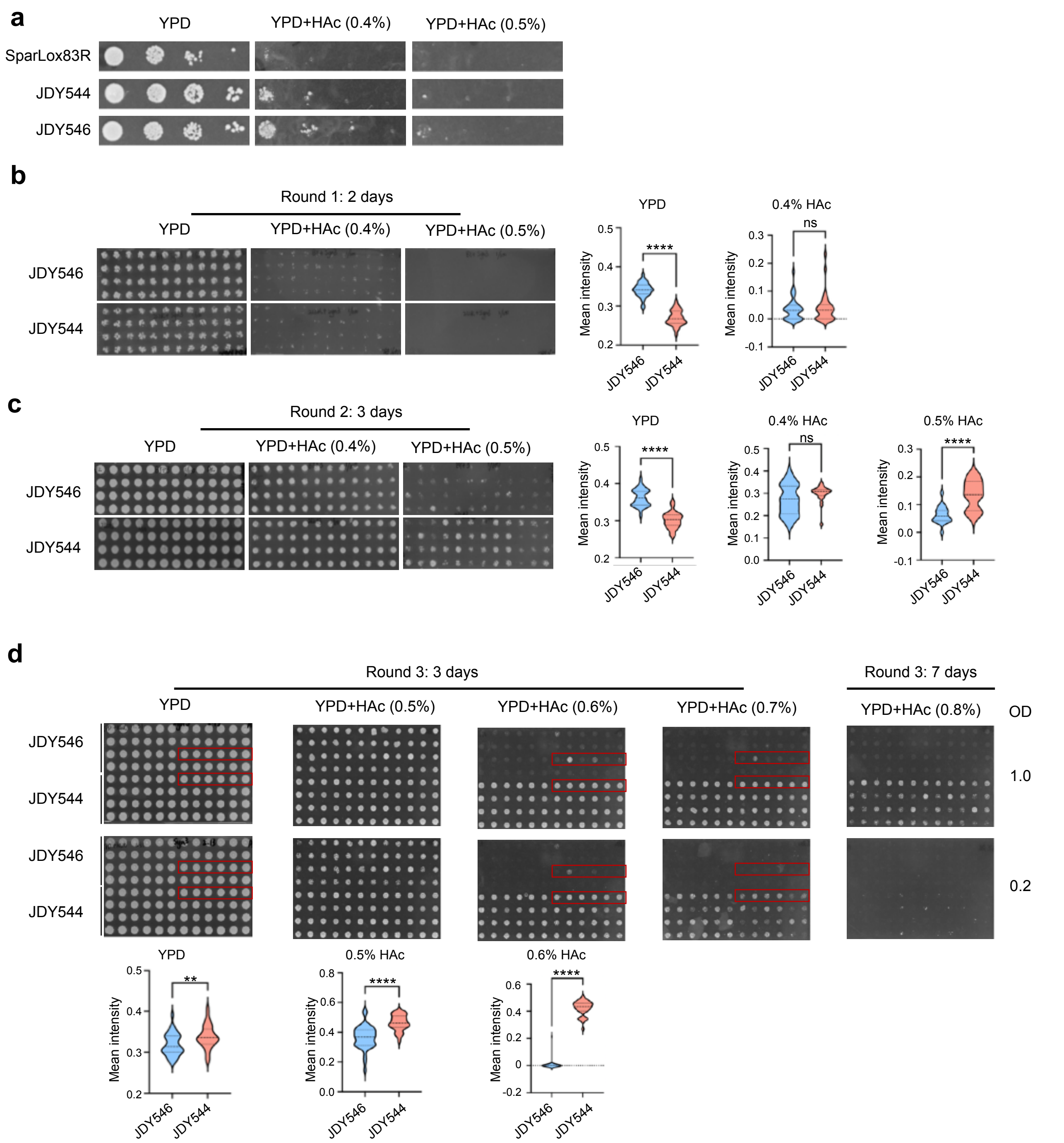

Supplementary Fig. 14 | Rapid development of HAc tolerance using whole-genome-wide SCRaMbLE in the presence of synIII or native chrIII. a. Serial dilution assay (10-fold) on YPD medium with/without HAc. Three strains, SparLox83R, BY4741 × synIII, and SparLox83R × synIII, all showed the expected growth defect on YPD with 0.4% and 0.5% HAc. **b&c**. Growth of 96 SCRaMbLEant populations after first and second round SCRaMbLE. After SCRaMbLE, the saturated culture was diluted 10- or 100-fold and spotted onto YPD with/without HAc. The growth of the strains were quantitatively evaluated using CellProfiler v4.2.6. An unpaired two-tailed t-test was used to determine statistical significance regarding differences in growth. ****p<0.0001. The bars show the mean and standard deviation of intensities of multiple colonies. **d.** Growth of 96 populations after third round SCRaMbLE. After SCRaMbLE, a 96-well plate reader was used to measure the density of all 96 populations. A_{600} values were adjusted to 1.0 and 0.2 and cells were spotted onto plates. Red boxes indicate the colonies shown in Fig. 6b. An unpaired two-tailed t-test was used. **p<0.01, ****p <0.0001. Source data are provided as a Source Data file. The exact p value was listed in Source data. Source data are provided as a Source Data file.

Supplementary Table 1 The numbers of rearrangement for strains in Figure 4.

Strain	Cre/loxPsym mediated rearrangement									Homologous recombination mediated rearrangement
	SynIII derived#							SparLox83R derived*	SynIII and SparLox83R derived*	
	Total	Del	Inv	Dup	Dup-inv	Inv-dup	Complex			
JDY549	95	66	7	/	/	14	8	1	0	0
JDY550	64	56	8	/	/	/	/	1	1	1
JDY551	88	63	5	/	/	20	/	1	0	0
JDY552	93	70	14	/	1	6	2	1	0	0
JDY553	90	63	7	12	1	7	/	0	0	0
JDY596	91	66	4	/	/	13	8	1	0	0
JDY597	92	63	9	/	/	20	/	1	0	0
JDY598	78	63	8	/	/	7	/	0	0	0
JDY599	89	63	13	/	/	13	/	1	0	0

#Each segment was considered as an individual unit for counting. Del, Deletion; Inv, Inversion; Dup, Duplication; Dup-inv, Duplicated inversion; Inv-dup, Inverted duplication.

***Rearrangements were counted by structural variatiant junctions**
