# Supplementary material for: Large-scale genomic rearrangements boost SCRaMbLE in Saccharomyces cerevisiae
Source: Nat Commun. 2024 Jan 26;15:770. doi: 10.1038/s41467-023-44511-5 (PMC10817965; doi:10.1038/s41467-023-44511-5)
Supplement: Supplementary file 3 — Description of Additional Supplementary Files [file 41467_2023_44511_MOESM3_ESM.pdf]

Title: Supplementary Data 1.
Legends: Locations of inserted loxPsym sites in SparLox83.

Title: Supplementary Data 2.
Legends: Sequences of edited sites

Title: Supplementary Data 3.
Legends: SNPs/InDels in SparLox83

Title: Supplementary Data 4. Legends: Primers used in this study.

Title: Supplementary Data 5.
Legends: List of strains used in this study.
