# Supplementary material for: Large-scale genomic rearrangements boost SCRaMbLE in Saccharomyces cerevisiae
Source: Nat Commun. 2024 Jan 26;15:770. doi: 10.1038/s41467-023-44511-5 (PMC10817965; doi:10.1038/s41467-023-44511-5)
Supplement: Supplementary file 4 — Supplementary Data 1 [file 41467_2023_44511_MOESM4_ESM.docx]

**Supplementary Data 1. Locations of inserted loxPsym sites in SparLox83**

| **Name** | **Insert site^a^** | **Notes** |  | **Name** | **Insert site^a^** | **Notes** |
| --- | --- | --- | --- | --- | --- | --- |
| I-1 | 51708 |  |  | VIII-1 | 74959 |  |
| I-2 | 115050 |  |  | VIII-2 | 345978 |  |
| I-3 | 209083 |  |  | VIII-3 | 489110 |  |
| II-1 | 56346 |  |  | IX-1 | 77997 |  |
| II-2 | 176659 |  |  | IX-2 | 411714 | Self-duplication^b^ |
| II-3 | 253209 | Off-target |  | IX-3 | 413059 |  |
| II-4 | 325134 |  |  | X-1 | 130456 | Self-duplication |
| II-5 | 433039 |  |  | X-2* | 131410 |  |
| II-6 | 479449 |  |  | X-3 | 188979 | Self-duplication |
| II-7 | 486400 |  |  | X-4 | 190016 |  |
| II-8 | 541862 | Off-target |  | X-5 | 303740 |  |
| II-9 | 563996 |  |  | X-6 | 554414 |  |
| II-10 | 648545 |  |  | X-7 | 642488 |  |
| II-11 | 671827 |  |  | XI-1 | 74611 |  |
| III-1 | 24306 |  |  | XI-2 | 356299 |  |
| III-2 | 200486 |  |  | XI-3 | 582098 |  |
| III-3 | 309197 |  |  | XI-4 | 635781 |  |
| IV-1 | 43937 |  |  | XII-1 | 83403 |  |
| IV-2 | 144091 |  |  | XII-2 | 133362 |  |
| IV-3 | 309055 |  |  | XII-3 | 234581 |  |
| IV-4 | 417044 |  |  | XII-4 | 419248 |  |
| IV-5 | 458920 |  |  | XII-5 | 447506 |  |
| IV-6 | 589165 |  |  | XII-6 | 782897 |  |
| IV-7 | 605638 |  |  | XII-7 | 924842 |  |
| IV-8 | 869600 | Off-target |  | XII-8 | 1090211 |  |
| IV-9 | 1029771 |  |  | XIII-1 | 119886 | Self-duplication |
| IV-10 | 1086043 |  |  | XIII-2 | 120927 |  |
| IV-11 | 1286916 |  |  | XIII-3 | 121977 |  |
| IV-12 | 1342001 | Self-duplication |  | XIII-4 | 123019 |  |
| IV-13 | 1342956 |  |  | XIII-5 | 762757 |  |
| V-1 | 70795 |  |  | XIII-6 | 804923 |  |
| V-2 | 78619 | Off-target |  | XIII-7 | 865447 |  |
| V-3 | 190095 |  |  | XIV-1 | 295627 |  |
| V-4 | 213214 |  |  | XIV-2 | 572843 |  |
| V-5 | 244493 |  |  | XIV-3 | 656031 |  |
| V-6 | 547928 |  |  | XV-1 | 216750 |  |
| VI-1 | 31506 |  |  | XV-2 | 743244 | Self-duplication |
| VI-2 | 218228 |  |  | XV-3 | 743745 |  |
| VII-1 | 196662 |  |  | XV-4 | 908241 |  |
| VII-2 | 245412 |  |  | XVI-1 | 451392 |  |
| VII-3 | 325056 |  |  | XVI-2 | 689950 |  |
| VII-4 | 888555 |  |  |  |  |  |

^a^Sequence coordinates are from the Saccharomyces Genome Database.

^b^Self-duplication: more than one loxPsym sequence separated by vector backbone.

*The X-2 loxPsym sequence has one mismatch.
