# Supplementary material for: Large-scale genomic rearrangements boost SCRaMbLE in Saccharomyces cerevisiae
Source: Nat Commun. 2024 Jan 26;15:770. doi: 10.1038/s41467-023-44511-5 (PMC10817965; doi:10.1038/s41467-023-44511-5)
Supplement: Supplementary file 5 — Supplementary Data 2 [file 41467_2023_44511_MOESM5_ESM.docx]

**Supplementary Data 2 Sequences of edited sites**

| **Site ID** | **Chromosome** | **Target sequence** | **Left homologous arm (HL)** | **Right homologous arm (HR)** |
| --- | --- | --- | --- | --- |
| I-1 | 1 | ATAAATGGCACGTGTATGTA | AAAAAACAAAAGCAAATCACATGTGCACATACGTCCAGAATGATATCAAG | CTGTGTAAATATGATAATCATCTCGGACGAACGGCGTAGCACTCTCCATC |
| I-2 | 1 | GCGACAAACATACATATTAT | TACAGTCGTGATACGATTTACTGTCACTTAGCAATAATATCTCGTACATA | ACACGTCGCCCTGAAAAAAAAAAACATAAGAAAAGAATACGAAAAAAAAG |
| I-3 | 1 | CCATTTCCAGTGCCTCCGAT | TTTGGCTTCCAGTATGCTTTCACGGAATTATTTCTCATGTACATTTAGCT | GAGGCATCATGGTACTACCGTGACGGAGAATACGTAGGCTGACTTTTTCG |
| II-1 | 2 | GAACGAACAGCAAGATAAAG | ACTTATCCATTATTTCCATCGTCAAAAAAAGGAAATAAATACTGTTGCTC | AAACGAAAAGTAAGCAGCTTCCTCAATATGCCGTCAAACGTACGTTCGGG |
| II-2 | 2 | ACGACATGGAAGCGATACAC | AGAGTTTGGTTGCCACATGTACGCTTAGGACACTCATGACTATCACTCCT | ATACCCACTGGCCGATTCTCACCCTCTGCATTGTATGATATTACTAGTAT |
| II-3* | 2 | N/A | N/A | N/A |
| II-4 | 2 | GACGGTAATGATTAGAGTTT | AAAAATTTAACGTTCGATAATTAAGTAACAACAGAGAAAATATGAGCTTA | TGTTTTATTTACTGTCACCTTGATGAGCGACTAAAAAGATAGAACGCGGG |
| II-5 | 2 | GCGAAAGTGAAAGGTGCAAG | TACATAAAATTATATATAAGAAACACTTTTGCTTTAGCCTTCCTTTCTTT | CGTCCTTTTTCACTCACAGCAACAAGCAGCAAGCACTAAGTACGCAGTCA |
| II-6 | 2 | TCATAATGGCGATGACAGGA | TGCTCGGACATGTTTGGGTGGACCTTATTCTACATGTTTTACTTTTCTCG | GCAAGCTCATCTCCCCAGCTTTAAAGGGTAGCTCAAGGAACACCTACTTC |
| II-7 | 2 | CACTGCGCTGAAATATTACG | CACTTACGCTGCAATAAGCAGCAACGGCATTGTAGTGATCATTTTCTACG | GAAAAAAATTGAAAATTTTTACTCTTCTCGAGTGTTGAATCACTGCTGCG |
| II-8* | 2 | N/A | N/A | N/A |
| II-9 | 2 | CTCAGTTTCAACATTATGAT | CTACTGCACTGTCATTATAGCCTAGTAAAGTATATAGTGAATACAATATA | TAACTCCATCAGAAAATATATTCATCGTCATATACGGAACATTCAGTTAT |
| II-10 | 2 | CTAAAGATAAAACTAACTGC | GAAGACATTAATACCTTTATTCATATAAGCACTTTCATTATCATTTTTTA | CACCAAGAAGTTCGAAGCCAAGAGTAAAGATGTCAGGAACCGGATTATCG |
| II-11 | 2 | CCCATATAGTGATGCCTAAG | GCCTTGCTCGTCATGAGAACGACTAACAAGTAAGAGCGCGATGTTGCTGT | GCCAGGAATGGCAAATTTACTTGATAAACTTCAGGCGATTGGATTTTGGT |
| III-1 | 3 | GTTGTTTGAAGCCCTTTAAA | GTAGCAAAGTTAATCTGCCAATTGACAGTAGTTTAATATATGGTATTATC | AAAAAACGGGTTAGGGCCACCCGGCGCGAAGTAATAGCTGCTGATTGGTC |
| III-2 | 3 | TCGAGAGTTTCACCAACCAT | TTGAACAAACGGCTGAGACGGGCAATACATATGCTCTACTTCTTTTCCAT | GCATACATTACCTTACGTGTGTTAGTGTACTATATTATATATATATATAT |
| III-3 | 3 | GGATTGATTATATAGGCATA | CATTTATCTTCATATTCATGAATTTCCTTACTGGACCCCCACCTTAGCAT | GCATTCCGTCCACTGTATCGTAGGATTATTTTCCAACATTAGTTAACTTA |
| IV-1 | 4 | ATATTCTAGCTTCGTTGTCA | TTCCTAATTGTGCATTTTTTCAATAACAATACTTATTCATCCTTATAATT | GAACATAGCCCATACACCGCAGTTATTTATGATCATTTCGAACGGGAAGT |
| IV-2 | 4 | GTTTAACGGTGCAGTGAGTT | TAGATAATCTTACAAGGGACAAGTAGTCAAGCCTTGCTTATTATAATTCT | ACATTCATGCAACGTGGTAAATATGTGTCTCTTTGCTTCTGTATTTAAGC |
| IV-3 | 4 | AATAAGTCAGCCCCTCCCTT | ATTTTTTTTTTTCATTTTTTAAAGGGTTTCTCTACAGCCTACAGGCCTCC | AGTGCGCTGTTGACCTGCGTATATAAGAGGTATATCAGTGCCAGTAGGTA |
| IV-4 | 4 | CGGTGAGAGGTGAGAGGTGA | AGTATAACAGTATATCTGACACGCACGTGATGACCACGTAATCGCATCGC | CTGACTCAGCTTCACTAAAAAGGAAAATATATACTCTTTCCCAGGCAAGG |
| IV-5 | 4 | GATAGAGCAGTACTTATATA | CGAAGCCCCTTATCCCCTAGTTACCGAAGAAGGCCACCAATCTTAAGTTT | CTATATATAGACTGGTTCACAAGGTTATCAATATGAAACTTGCGCGATCA |
| IV-6 | 4 | CTATATCATAGCCAGTTAGC | AAAAAGCTACGCAAATATCGTATATCTGTTATACTACAAAACAATTACTT | AACGACTTCAGCTAAATGGACTATCCATGCTTTAGGCAGAGGCGAAGCGC |
| IV-7 | 4 | CAGAAGTAGATAAAGCAGCC | AAAAAAACAGGTAGAAGAAGGCTTGCTATAATTTGAACACTCTCTACCCT | TTGGCTTGAGAAACGTCATATCTATATATAGCGTAGATATGTTTATTCGC |
| IV-8* | 4 | N/A | N/A | N/A |
| IV-9 | 4 | GGCCTTGATGTTATTAGTAC | TGGAAAAATATGACATAAGGTATGCGTATTAGTAAACTATTAGGGAGCCG | ATCTCACGCCGAGACTTACTTGGACTTTTCTCTATTGTAAAGCGGAAAAA |
| IV-10 | 4 | CATCCTTACTACTTTCCTCG | TTTTCGTTTCGCAGCGAATCCCTTTTAGCAGAGGAAAAAAAAGATGAAAC | AGACTCAACAGTAAAGGTTACTTTCAATTCAATAAACAAAAGGCACAGCG |
| IV-11 | 4 | AAAGGGAGCATGTACATCTG | GGGAAAAGAATACTGCTACTGCTGTGCGAGACTTTGGTAGTAGGGATCGG | AATATATAAGCAGGAGCTCTCTACCTGGACCAAATTGCCTTCTTATGTTG |
| IV-12, 13 | 4 | TCTCAGCATAGCATTAACAT | CGTAAGAAATACACATATAGTAGGTTTTGTCGCCTCCTCTTCCCCCTTTG | TGCCTCAGAGTGACAAAGAGAGAAATAGTTAACTAGAATACGGTGCAAAT |
| V-1 | 5 | AGCGACTAACTACCCTATTA | CTAATTATGTCGGCACATTTGAGAACCAATGGTTAGTTTCCAGCGCACCT | AGGGACGAAGGTGGTCTTTCTGAGGGAAGGAGGAAAAAAAGGTAAGAACC |
| V-2* | 5 | N/A | N/A | N/A |
| V-3 | 5 | CAAAGAGAGCATGTCCATAG | CTACTCATTCAAAAATTATCCCTCTTCACTTCCCGTATTCACACTTGTTG | AAAAATGTAGTCTCACCCACAGAAAAGAAAAGATCATTTGAAAATAAGAT |
| V-4 | 5 | ACCCATGTGAGACTGAAACA | GAAAACCTTTGGAGAAAATCATTGCAAATTTAAAAGCTGTGCTTCAAAAA | TCCAGTGGTCTTCATCCGGACCGGTTCAAAGTCCTGCTCTACCTTCAATA |
| V-5 | 5 | TCTTAGATGCAGATATTCTT | TATAATTAGTTTTTCATCTGAAAGATATTTAGGGCACCATTTTCTTTTGT | CGATTTACCCTGGTGGTACAGAAGATTATGTTACATAATTCATCAATTTT |
| V-6 | 5 | GGCAAATAGCTTCCTCTTTG | TTTGCGGAAGCTACTTTATTCCGGCCTGGAGTCAAAAGAGGAAGCTCGGT | CCGGGGCGCGGGGGGACGAGGCAAAAAGCAAAGAAAAGCAAAAAAAATAA |
| VI-1 | 6 | CCTTGCTGAACATTGAATAG | TAGAGGTTTATATTATAAAAGTGGAAAGGTAAAATCAACAGCGTTTATTT | ACGGTAGAGACTACTATTGCTAAACAATTACTTACATGGAAATGTACTGT |
| VI-2 | 6 | GTAATCACTATAAACGCGTA | AAGCATTGCTTCATGGAGGGGGTTGACTTCTTGAATAAAATGGCTTTCTG | CGTGTTGCGTGGCTCTGATGATGGGCATTTCTAATTTTAAGATCAACAAC |
| VII-1 | 7 | ACGTCAAGTGAGAAGAGTTT | TTTAGTGAAGAAAGAGAAAAGTTGCTGCTCTAGATTTTGTATCGGCTATT | CCTTCGCTTCAGTTAGATCTTCTATTATTTCCTTTTTTTTCTTTTTGTTT |
| VII-2 | 7 | GAAGACCTATCAATTTTATG | TTTCTTTCTTTAGACATCAAACTGGTAGTTCTTATCAGTCTCAGCCTTTT | AGGGTAATGGTTGCTTCCTTTTCCCTAGCAGCGGCCCATCGCTTTAGCTT |
| VII-3 | 7 | ACTTATTAGTAAGAAGAGCA | TTCCGACTAAAACCCGCCTTCCCACGCGAAATTCTGGGCCGTTCAAGGCA | GGAAGGTACATTAAAGCAAGAGAACCGTGCATGAATTTATAGACATTTTT |
| VII-4 | 7 | AGCAAAAGTAGATCATTCAC | ATAAAACATAAACAAAAAAGAAAAATTAAGATTTGCAATTCTGCCGCTTA | AATATATATATATAAACGCATTTATAATCTTGTAACGTGCACTCAATTTA |
| VIII-1 | 8 | CACATCTTGCCTGTTATCTA | ACCTATGGGAAAGGTGTAACTCATCATTGCGCTTTCTACGGTACGGTTAT | CTCTATTCATCGGTGTTGCATGAAGATACGTCTTGTTCGCCAAGTATATA |
| VIII-2 | 8 | ACCTTAGCATTGGGATCAGC | GAGAGAATATCACTGTTTACGGCCCACAAAGAGAATGAGAAAATTGATAA | AGCCTTAGAATTCTGGGTGTGAACAGAAACACATTAGTGCATTAGTGTAT |
| VIII-3 | 8 | GAGCAGCGAGAACACGACCA | CTGCACTTTGCATCGGAAGGCGTTATCGGTTTTGGGTTTAGTGCCTAAAC | GCTATATAAATGGAAAGTTAGGACAGGGGCAAAGAATAAGAGCACAGAAG |
| IX-1 | 9 | GGCGCTATCAAAGGGAAACG | AACTTGACGCGTCAACATGAGGAGGGTAATGATGTGGTAGCGCCGTGTAA | ATAATAGTATTAACACCGCAGCTTTTTTTTCCTTTCTCCCTCTATTGGTT |
| IX-2 | 9 | CCAGGCAGAGTTGTGAAACC | AAATCAAAATAAACATCAAAAGAACCGCCAATTGATAAAAGCACAAAGTA | CCTCTTCTATTGTACCACACACAAATTTTTCTTTATTTAGGTAGAGGATG |
| X-1, 2 | 10 | TAGTAAAAGAAACGTCGATG | AATTATACCAACATGGTTGTAGCATTTCAAGTTAGCTTGTTCGAGCTGTA | CTAATTTGTTCCGGTGACTTTCAGGTCACGTGTTTTCCATGCGTGCGTTG |
| X-3, 4 | 10 | ACTCTCAACAGTGATATTTC | AACAACTCATTACTAGACTACTACCAGTTACTACACATCGATACAACTCC | TCATGTCAAGACGTAGTATAGTATTGCAACAGGAAAAAAAAATCTTATTG |
| X-5 | 10 | CAACGGACCCTCTTAATTAG | GGTTTTTTTCGCCTCTCTTCAAGTTTCCTATACCCGAGCTTAACACAACA | TATCCTCCGTTCTTTTCTTCCGCTTTCCTAATCGAGATACCAGAGCACGG |
| X-6 | 10 | GATATGTCTTGCAAAACTAT | CCTCCCCGTTTGTATCAAAAGTATCCGAAACTGTTCTATGCGTGCAAAAT | AAAGACCCTTCCACTACAGGTACAGTTTAGAAATGCCGGAGCTGCAAGTA |
| X-7 | 10 | CACCACTGTCTTCTTTCCTG | AATCCCCTAAACATTCAGATTGTAAACTAGGGTTGAGAAAATGACTCATC | CATTCTATAGATTATTGTGAATGACTCTTATTGATGAGATGGCAATAACT |
| XI-1 | 11 | CATGGATATTAGAGGATAGT | CATTATTTATATCATATTCTTACACTTCATACAATTATATATACTCGTAC | TTTTTGTTTAAACTAATTCTATGTAACCTCGATGGCATGAGTCCATAATA |
| XI-2 | 11 | ACGATGCGATGATAAATATT | ACATGCAAAATCAGCCCACTCGAAGTTAGATACTGCGTAGACGGATTTGC | GACAGTGCGTGCCCACCCATAAGTTGCTGTAGAGCTTACAAATTGTAAAG |
| XI-3 | 11 | GAAAAGCCTCAACTATTTAT | TGTAGTGACGCGGAAACGTTTTACTCGTCAATGGACATTTGCGAATGTGT | CTGCATTCTGTCAAGAAGGGTATGTGTATGAACATGCAAATGACACTGTA |
| XI-4 | 11 | TTGTGCGCTGAGTAATCATT | AAAAAAGGGATTGACATTTCTTCGAGAATTAGTTGAGAAACCCTTTTAGA | TTGATTATAAGCTCTGAAAGGTTACTGCTACTGCTAGTAGTCTTTCAGGA |
| XII-1 | 12 | GAATGAGGGATGGAACAAGA | CCCCATTTTCCCGAATTTTCTTCTACGTTTTCTTTTTTCAGAAATACTTC | AAGCGACATACAAAAACTGAGTCAGTTTAGTCTTAATAGGTGCAATATTT |
| XII-2 | 12 | GAAGTGGTGTAGTAGTGATG | TATAGTATATACTACCTGTAAATATGTGCGATGCACAATTAACATTACCT | TACTGCTAGCACTGTCCTCTTGTGCTTGGCCCCTTAAGAGTGTTCTAAGA |
| XII-3 | 12 | TACTGCACTGTCACTTACCA | AAGTTTTATAAGCATTTTTATGTAACGAAAAATAAATTGGTTCATATTAT | AAAGACCAGACAAGAAGTTGCCGACAGTCTGTTGAATTGGCCTGGTTAGG |
| XII-4 | 12 | TCTTCTTGACAGCACTGCCT | AATGCCATTCTGCCTAGCCCATTTGCGTCTTCGCTGCCGTAACCATTCTC | TCAGTTCTCTATATTTGTCGCGCCGCCGCAAAATGCACCCACATTAAAGA |
| XII-5 | 12 | GATGCATTACGAGAAGGTTA | TTGTTCGTTGTGCACGTAGGATGTATATTGAACAAGCATGACCAGAATCT | GATGATATCAGACCTCCGAAGTCCATGTTGCAAAATGTGCCGACTTTCCG |
| XII-6 | 12 | AATTTAAAACCGTGGCTTGC | GCCGCAAATACAGAGGCGCCCCAGACAACACCGCAGTGTGAAGCACTGTC | AGATGCCCAGACCAACCCTGTTGGGTTTTTCTCTCGAGCACGCCGTTATA |
| XII-7 | 12 | TTGCATACAAATCCCTCTGA | AGTGGAAAACGACATAAATAATAATTTTATAATATAATAATGATAATTCA | ATGATTGATGACGCGAGAAAAAAAAACGCGAAATTTTTCTTCCCAAAGCT |
| XII-8 | 12 | CTACTTGTCGATGCATATAT | ATAAAAAAGCAATATTTTTTTGCGAGCTATTTAGTGATATAGCCGCCCAG | CTGAGAGTACTAATACAATTATCGGAAGACCTCAAAGTAAAATATAGAAG |
| XIII-1, 2, 3, 4 | 13 | CGTTAAACGCTGCTGATTGA | CATTAGAAATAAGGCTCTCGTTGATCATCCTTGTAACTGAAAATTAGAAC | ATTCCAGCCGTTCTCTTCCAACCCCTTTTACCCCGATTGTTCGTCCACTA |
| XIII-5 | 13 | CAACCATCTTCGCCAAGTAG | ATCATACTTAGTAACAAGAAAGACAAAAGCGCAAACCGAACCGCCCAGCT | CAAACTTATATAAGCTCCAACGATGTCCCCATCAATTAAGAACCCTCGAT |
| XIII-6 | 13 | GTTTAGCTGTTATCCTCTGA | GTTCGGCAGTCTAAAGGTTAAACATTATCAGAAATTATCTATTCCTATTT | TTGAATTGTAATTTTACTAAGAAAAGGAGAGGAAAAGGACGTGCATATAT |
| XIII-7 | 13 | CTTTATCCATCATTGAGACA | CGTGCTTTTATTAGTGGGCCCCTTCTTTGAGACCCCGCGGGTGATATGGC | TTGTTGGAAGCAATTACCAAGACAATCACAAACGAAGGTTGCTACCGAAG |
| XIV-1 | 14 | AATAGTTTGAGCCAGCACGA | CGATGATAAATCCTCCGCCGCATGATGCTTTTGATTTGCCTAAGGGCCTG | GAGGGTCAACATACCTTGAAAATCCAAGTAAAAGGATGGATATCGTTATA |
| XIV-2 | 14 | ATATATAGCTCGCTCATGCA | TGAGAGGGTAGGCAAGAAGGTCGAAAGGAAAACAAAAAAACGTATCGCTG | CACAAATACAGGGGTAGGGTCCTCGACGTAGTAGACATTCTGCTTCTATA |
| XIV-3 | 14 | CTGTGACGTCGATGCATGCG | GACACTTATACTTGGTGGGGAATCGCCCGTCAGGCCTGAACGCAACGAAC | TGAGCTCAGGCCGCATCACGGCCGTTACGCCCTCCAGAGTCACCACGACT |
| XV-1 | 15 | CGATATGATGGTGATGGTGA | AGCAATTCGGGAGGGCGAAAAATAAAAACTGGAGCAAGGAATTACCATCA | CCTTAGCCTCTAGCCATAGCCATCATGCAAGCGTGTATCTTCTAAGATTC |
| XV-2, 3 | 15 | CTGTGAGCAGTAATTATCAA | AACCATGAAAATATGGTCTCCAGGTTATCAATAGCTTCGAATTGGAAATA | ATGCTATTATATAAATATACATACCTACACCCATCCCATATTTACATAGA |
| XV-4 | 15 | GCTCTATCGGTCCGCTAGCT | GTAAGACCGATCCACTTTGCCAGCTGCTTACGCTGCGGAAAGTAAACAGA | CGGCTGAACTTTATAACAAATGCGCCTTCTAACAAGCGATGAAGCCATGC |
| XVI-1 | 16 | GTGGACGGTTCTTAAAATTC | GGATTGTTTACCAGCCGGCAGGACTCTGTTGATTTGTTTCACCTGTGATG | CACCTGTTAATAATATAATTGTGCAAATGCGCGCTTTTTTCGCCGCTCAC |
| XVI-2 | 16 | ATAACCACCATGTCAGCACC | TCCTGCTGGTGTCGTATTATTTTTTGAAATTATTTTTCAATAACCACCAT | TTCTTTTACAATTATACAAACACACATATCTCAAAATCACTCAAGAGGTC |

*Off-target site.
