# Supplementary material for: Large-scale genomic rearrangements boost SCRaMbLE in Saccharomyces cerevisiae
Source: Nat Commun. 2024 Jan 26;15:770. doi: 10.1038/s41467-023-44511-5 (PMC10817965; doi:10.1038/s41467-023-44511-5)
Supplement: Supplementary file 7 — Supplementary Data 4 [file 41467_2023_44511_MOESM7_ESM.docx]

**Supplementary Data 4 Primers used in this study.**

| **Name** | **Sequence** | **Description** |
| --- | --- | --- |
| MGO001 | CACCTTACTGGAATTTAGTCCCTGCTA | Used to verify loxPsym insertion, paired with site-specific primers. |
| MGO002 | CTTCGCCAACTGCAACGGAATA | Used to verify duplication. For amplicon 1 in Extended Data Fig 4a. |
| MGO003 | GGCGAATTTGACACAGCTAGTAAGG | Used to verify duplication. For amplicon 1 in Extended Data Fig 4a. |
| MGO004 | GGCAGCATCACCAATCAATCTTTCT | Used to verify duplication. For amplicon 2 in Extended Data Fig 4a. |
| MGO005 | GCGGAAACCAGCGTCACTAATTT | Used to verify duplication. For amplicon 2 in Extended Data Fig 4a. |
| MGO006 | CTTAATGGAGATTGAGGCAGCAAT | Used to verify translocation. For amplicon 3 Extended Data Fig 4a. |
| MGO007 | GTCATCCTCCCTGTGTTTACAGTG | Used to verify translocation. For amplicon 3 Extended Data Fig 4a. |
| MGO008 | TTCTATAGAACGTGTATGGCTCACG | Used to verify translocation. For amplicon 4 Extended Data Fig 4a. |
| MGO009 | GCAGGTGTTAATAGAGATGAAGGTAGATCC | Used to verify translocation. For amplicon 4 Extended Data Fig 4a. |
| MGO010 | CTCTTCACCAATCAGGGACATATCAGTACT | Used to verify inversion. For amplicon 5 in Extended Data Fig 4a. |
| MGO011 | CGCTATCTCAAGGCAAACGTTCT | Used to verify inversion. For amplicon 5 in Extended Data Fig 4a. |
| MGO012 | CCGAGCTAATTTCAAGTGGGTGAC | Used to verify inversion. For amplicon 6 in Extended Data Fig 4a. |
| MGO013 | CTGTACATACCGAAACGCCATCA | Used to verify inversion. For amplicon 6 in Extended Data Fig 4a. |
| MGO014 | TTCCTTCTTGTCGACGACAGGTTTC | Primer A in Extended Data Fig 9b. Used to verify translocation in JDY528 strain. |
| MGO015 | CAGGCGTTCTTGTATCTAGTAATCTCCT | Primer B in Extended Data Fig 9b. Used to verify translocation in JDY528 strain. |
| MGO016 | ATTTGGAAAATTTACCACTGCCCATGG | Primer C in Extended Data Fig 9b. Used to verify translocation in JDY528 strain. |
| MGO017 | CTTAATGGAGATTGAGGCAGCAAT | Primer D in Extended Data Fig 9b Used to verify duplication in JDY528 strain. |
| MGO018 | GCACATTGAATTTACACTCCCGATC | Primer E in Extended Data Fig 9b. Used to verify duplication in JDY528 strain. |
| MGO019 | ACCCTTACCATCGCAGGACTTTTCAGTC | Primer R1 used in Extended Data Fig 12a. |
| MGO020 | CGGTACTACCAGCGTTTTGTTGTTGGTC | Primer F1 used in Extended Data Fig 12a. |
| MGO021 | TAGCGTCTTTCATCGAGGTAGCGTTTGC | Primer R2 used in Extended Data Fig 12a. |
| MGO022 | GTTGCCGAAGAGACACCAAAATGTGCC | Primer F2 used in Extended Data Fig 12a. |
| MGO023 | GATACAAATGCCCCGGAGAATCTAGTGTACC | Primer R3 used in Extended Data Fig 12a. |
| MGO024 | AACAACTCGGCTGCCGTCTGGGCTATAA | Primer F3 used in Extended Data Fig 12a. |
