# Supplementary material for: Large-scale genomic rearrangements boost SCRaMbLE in Saccharomyces cerevisiae
Source: Nat Commun. 2024 Jan 26;15:770. doi: 10.1038/s41467-023-44511-5 (PMC10817965; doi:10.1038/s41467-023-44511-5)
Supplement: Supplementary file 8 — Supplementary Data 5 [file 41467_2023_44511_MOESM8_ESM.docx]

**Supplementary Data 5. List of strains used in this study**

| **Systematic Name** | **Mating type** | **Genotype** | **Description** |
| --- | --- | --- | --- |
| BY4741 | a | *leu2∆0 met15∆0 ura3∆0 his3∆1* |  |
| BY4742 | alpha | *leu2∆0 lys2∆0 ura3∆0 his3∆1* |  |
| JDY524 | a | *his3∆200 leu2∆0 lys2∆0 trp1∆63 ura3∆0 met15∆0 ho:: pTDH3-Cas9-tCYC1-TRP1 can1::PED* | Initial strain, with Cas9 gene inserted into *HO* locus. |
| SparLox83 | a | *his3∆200 leu2∆0 lys2∆0 trp1∆63 ura3∆0 met15∆0 ho:: pTDH3-Cas9-tCYC1-TRP1 can1::PED* | Final strain, with 83 loxPsym sites inserted across all 16 chromosomes. |
| SparLox83R | a | *his3∆200 leu2∆0 lys2∆0 trp1∆63 ura3∆0 met15∆0 ho:: pTDH3-Cas9-tCYC1-TRP1 can1::PED ReSCuES[URA3-leu2]* | ReSCuES sequence inserted into SparLox83 at an intergenic region between *YFR015C* and *YFR016C* on chrVI. The *URA3* gene is in frame and the *LEU2* gene is not in frame. |
| JDY528 | a | *his3∆200 leu2∆0 lys2∆0 trp1∆63 ura3∆0 met15∆0 ho:: pTDH3-Cas9-tCYC1-TRP1 can1::PED ReSCuES[LEU2-ura3]* | Strain picked from nocodazole containing medium after SCRaMbLE induction in SparLox83R. The ReSCuES sequence is inverted so that the *LEU2* gene is in frame and the *URA3* gene is not in frame. |
| JDY529 | a | *his3∆200 leu2∆0 lys2∆0 trp1∆63 ura3∆0 met15∆0 ho:: pTDH3-Cas9-tCYC1-TRP1 can1::PED ReSCuES[LEU2-ura3]* | Strain picked from nocodazole containing medium after SCRaMbLE induction in SparLox83R. The ReSCuES sequence is inverted so that the *LEU2* gene is in frame and the *URA3* gene is not in frame. |
| JDY530 | a | *his3∆200 leu2∆0 lys2∆0 trp1∆63 ura3∆0 met15∆0 ho:: pTDH3-Cas9-tCYC1-TRP1 can1::PED ReSCuES[LEU2-ura3]* | Strain picked from rapamycin containing medium after SCRaMbLE induction in SparLox83R. The ReSCuES sequence is inverted so that the *LEU2* gene is in frame and the *URA3* gene is not in frame. |
| JDY531 | a | *his3∆200 leu2∆0 lys2∆0 trp1∆63 ura3∆0 met15∆0 ho:: pTDH3-Cas9-tCYC1-TRP1 can1::PED ReSCuES[LEU2-ura3]* | Strain picked from rapamycin containing medium after SCRaMbLE induction in SparLox83R. The ReSCuES sequence is inverted so that the *LEU2* gene is in frame and the *URA3* gene is not in frame. |
| JDY532 | a | *his3∆200 leu2∆0 lys2∆0 trp1∆63 ura3∆0 met15∆0 ho:: pTDH3-Cas9-tCYC1-TRP1 can1::PED ReSCuES[LEU2-ura3]* | Strain picked from benomyl containing medium after SCRaMbLE induction in SparLox83R. The ReSCuES sequence is inverted so that the *LEU2* gene is in frame and the *URA3* gene is not in frame. |
| JDY536 | a/alpha | *his3∆200/his3∆1 leu2∆0/leu2∆0 lys2∆0/lys2∆0 trp1∆63/TRP1 ura3∆0/ura3∆0 met15∆0/MAT15 ho:: pTDH3-Cas9-tCYC1-TRP1 can1::PED ReSCuES[URA3-leu2]* | SparLox83R crossed with BY4742 |
| JDY541 | alpha | *his3Δ1 leu2Δ0 lys2Δ0 ura3Δ0 synIII HO::synSUP61* | Strain with complete *synIII* |
| JDY543 | a | *leu2∆0 met15∆0 ura3∆0 his3∆1 ReSCuES[URA3-leu2]* | ReSCuES was inserted into BY4741 at an intergenic region between *YFR015C* and *YFR016C* on chrVI. The *URA3* gene is in frame and the *LEU2* gene is not in frame. |
| JDY544 | a/alpha | *his3∆200/his3Δ1 leu2∆0/leu2∆0 lys2∆0/lys2∆0 trp1∆63/TRP1 ura3∆0/ura3∆0 met15∆0/MAT15 ho:: pTDH3-Cas9-tCYC1-TRP1/ HO::synSUP61 can1::PED/CAN1 ReSCuES[URA3-leu2]* | SparLox83R crossed with JDY541 |
| JDY546 | a/alpha | *leu2∆0/leu2∆0 met15∆0/MAT15 ura3∆0/ura3∆0 his3∆1/ his3∆1 HO::synSUP61 ReSCuES[URA3-leu2]* | JDY543 crossed with JDY541 |
| JDY549 | a/alpha | *his3∆200/his3Δ1 leu2∆0/leu2∆0 lys2∆0/lys2∆0 trp1∆63/TRP1 ura3∆0/ura3∆0 met15∆0/MAT15 ho:: pTDH3-Cas9-tCYC1-TRP1/ HO::synSUP61 can1::PED/CAN1 ReSCuES[LEU2-ura3]* | Strain selected after five SCRaMbLE rounds with JDY544. |
| JDY550 | a/alpha | *his3∆200/his3Δ1 leu2∆0/leu2∆0 lys2∆0/lys2∆0 trp1∆63/TRP1 ura3∆0/ura3∆0 met15∆0/MAT15 ho:: pTDH3-Cas9-tCYC1-TRP1/ HO::synSUP61 can1::PED/CAN1 ReSCuES[LEU2-ura3]* | Strain selected after five SCRaMbLE rounds with JDY544. |
| JDY551 | a/alpha | *his3∆200/his3Δ1 leu2∆0/leu2∆0 lys2∆0/lys2∆0 trp1∆63/TRP1 ura3∆0/ura3∆0 met15∆0/MAT15 ho:: pTDH3-Cas9-tCYC1-TRP1/ HO::synSUP61 can1::PED/CAN1 ReSCuES[LEU2-ura3]* | Strain selected after five SCRaMbLE rounds with JDY544. |
| JDY552 | a/alpha | *his3∆200/his3Δ1 leu2∆0/leu2∆0 lys2∆0/lys2∆0 trp1∆63/TRP1 ura3∆0/ura3∆0 met15∆0/MAT15 ho:: pTDH3-Cas9-tCYC1-TRP1/ HO::synSUP61 can1::PED/CAN1 ReSCuES[LEU2-ura3]* | Strain selected after five SCRaMbLE rounds with JDY544. |
| JDY553 | a/alpha | *his3∆200/his3Δ1 leu2∆0/leu2∆0 lys2∆0/lys2∆0 trp1∆63/TRP1 ura3∆0/ura3∆0 met15∆0/MAT15 ho:: pTDH3-Cas9-tCYC1-TRP1/ HO::synSUP61 can1::PED/CAN1 ReSCuES[LEU2-ura3]* | Strain selected after five SCRaMbLE rounds with JDY544. |
| JDY596 | a/alpha | *his3∆200/his3Δ1 leu2∆0/leu2∆0 lys2∆0/lys2∆0 trp1∆63/TRP1 ura3∆0/ura3∆0 met15∆0/MAT15 ho:: pTDH3-Cas9-tCYC1-TRP1/ HO::synSUP61 can1::PED/CAN1 ReSCuES[LEU2-ura3]* | Strain selected after five SCRaMbLE rounds with JDY544. |
| JDY597 | a/alpha | *his3∆200/his3Δ1 leu2∆0/leu2∆0 lys2∆0/lys2∆0 trp1∆63/TRP1 ura3∆0/ura3∆0 met15∆0/MAT15 ho:: pTDH3-Cas9-tCYC1-TRP1/ HO::synSUP61 can1::PED/CAN1 ReSCuES[LEU2-ura3]* | Strain selected after five SCRaMbLE rounds with JDY544. |
| JDY598 | a/alpha | *his3∆200/his3Δ1 leu2∆0/leu2∆0 lys2∆0/lys2∆0 trp1∆63/TRP1 ura3∆0/ura3∆0 met15∆0/MAT15 ho:: pTDH3-Cas9-tCYC1-TRP1/ HO::synSUP61 can1::PED/CAN1 ReSCuES[LEU2-ura3]* | Strain selected after five SCRaMbLE rounds with JDY544. |
| JDY599 | a/alpha | *his3∆200/his3Δ1 leu2∆0/leu2∆0 lys2∆0/lys2∆0 trp1∆63/TRP1 ura3∆0/ura3∆0 met15∆0/MAT15 ho:: pTDH3-Cas9-tCYC1-TRP1/ HO::synSUP61 can1::PED/CAN1 ReSCuES[LEU2-ura3]* | Strain selected after five SCRaMbLE rounds with JDY544. |
