# Supplementary material for: Large-scale genomic rearrangements boost SCRaMbLE in Saccharomyces cerevisiae
Source: Nat Commun. 2024 Jan 26;15:770. doi: 10.1038/s41467-023-44511-5 (PMC10817965; doi:10.1038/s41467-023-44511-5)
Supplement: Supplementary file 9 — Reporting Summary [file 41467_2023_44511_MOESM9_ESM.pdf]

nature portfolio

Corresponding author(s): DBPR, NCOMMS-23-29049A

Last updated by author(s): Dec 10, 2023

Reporting Summary

Nature Portfolio wishes to improve the reproducibility of the work that we publish. This form provides structure for consistency and transparency in reporting. For further information on Nature Portfolio policies, see our Editorial Policies and the Editorial Policy Checklist.

Statistics

For all statistical analyses, confirm that the following items are present in the figure legend, table legend, main text, or Methods section.

n/a	Confirmed	
[ ]	[x]	The exact sample size (*n*) for each experimental group/condition, given as a discrete number and unit of measurement
[ ]	[x]	A statement on whether measurements were taken from distinct samples or whether the same sample was measured repeatedly
[ ]	[x]	The statistical test(s) used AND whether they are one- or two-sided<br>*Only common tests should be described solely by name; describe more complex techniques in the Methods section.*
[x]	[ ]	A description of all covariates tested
[x]	[ ]	A description of any assumptions or corrections, such as tests of normality and adjustment for multiple comparisons
[ ]	[x]	A full description of the statistical parameters including central tendency (e.g. means) or other basic estimates (e.g. regression coefficient) AND variation (e.g. standard deviation) or associated estimates of uncertainty (e.g. confidence intervals)
[ ]	[x]	For null hypothesis testing, the test statistic (e.g. *F*, *t*, *r*) with confidence intervals, effect sizes, degrees of freedom and *P* value noted<br>*Give P values as exact values whenever suitable.*
[x]	[ ]	For Bayesian analysis, information on the choice of priors and Markov chain Monte Carlo settings
[x]	[ ]	For hierarchical and complex designs, identification of the appropriate level for tests and full reporting of outcomes
[ ]	[x]	Estimates of effect sizes (e.g. Cohen's *d*, Pearson's *r*), indicating how they were calculated

Our web collection on statistics for biologists contains articles on many of the points above.

Software and code

Policy information about availability of computer code

Data collection	For nanopore sequencing, genomic DNA was extracted according using a modified Qiagen Genomic-tip 100/g protocol with the Qiagen Genomic Buffer kit. DNA quality was assessed with agarose gel electrophoresis and with a NanoDrop™ 2000 Spectrophotometer and a Qubit 4 Fluorometer with dsDNA HS reagent. Oxford Nanopore Technology (ONT) sequencing library preparation was performed using SQK LSK108 or LSK109 kits with Native Barcoding kits EXP-NDB104 or EXP-NBD114, according to the manufacturer's guidelines. Sequencing was performed on a MinION Mk1B device using FLO-MIN106D with R9.4.1 sequencing cells. Sequencing was performed for 48 hr using MinKNOW (v3.6.5) software.<br>Hi-C library preparation was performed at Beijing Novogene Bioinformatics Technology Co. Ltd. Samples were flash-frozen and pulverized before formaldehyde crosslinking. Cell lysis and MboI digestion were performed after crosslinking, then DNA ends were labeled with biotin and joined. DNA was decrosslinked using Protease K and SDS. Free DNA was purified and extracted using Ampure XP beads. After quality control, ultrasonication was used to produce 200–500 bp DNA fragments. The biotin-labeled fragments were further enriched and sequenced by Illumina Novaseq.<br>For PFGE, samples were analyzed using a 0.9% agarose gel with 0.5× TBE (pH8.0) for 20 hrs at 14 °C on a BioRad CHEF Mapper apparatus. Voltage was 6 V/cm, at an angle of 120°, with 10–60 s switch time.<br>For RNA seq, SparLox83R and JDY528 strains, each with 3 biological replicates, were cultured overnight in YPD medium at 30°C. Subsequently, they were re-inoculated in 50 mL fresh YPD medium for further incubation until OD600 reached approximately 0.6. The cells were treated with DMSO or nocodazole at a concentration of 10 μg/mL for 2 hours prior to harvesting. The RNA sequencing experiments were conducted at Beijing Novogene Bioinformatics Technology Co., Ltd. Libraries were prepared using the non-stranded illumina NEBNext UltraTM RNA Library Prep Kit.
Data analysis	All possible combinations of the 83 loxPsym sites were assembled in silico using the yeast reference genome (GCF_000146045.2). Only 1kb-long loxPsym neighboring sequences were retained. The assembled sequences were used as the reference for junction detection. LoxPsym-containing ONT sequence reads were identified using LAST software (v1250) and then aligned to the reference using Minimap2 (v2.20-r1061). Reads with MapQ > 20 that covered at least one reference sequence were retained for subsequent analysis. Reads containing more than one loxPsym site were analyzed separately. The Rearrangement Rate (RR) of a particular loxPsym site, i, was defined as Nrei/(2Nnormi+Nrei), where Nnormi and Nrei were the number of normal and rearranged reads for loxPsym site i. The Rearrangement Rate (RR) was subsequently normalized to the total number of rearranged loxPsym sites to calculate Average Rearrangement Rate (ARR). The Rearrangement Weight (RW) of two particular loxPsym sites, i and j, was calculated by equation 1, which also considered bias of sequencing depth.<br><br>Networks of inter-chromosomal and intra-chromosomal rearrangements were constructed using Cytoscape software (v3.7.1).<br><br>Assembly-based structural variant calling: ONT reads (FASTQ format) were used with the Canu pipeling (v2.1.1) to assemble the SparLox83R and JDY528 genomes. Subsequently, Pilon software was employed to correct draft assemblies, utilizing the high-quality Illumina PE300 reads, for three consecutive rounds. The assembled genomes of JDY528 was aligned to SparLox83R using Minimap2 (v2.20-r1061) software with -x asm5 argument. The resultant PAF format files were used to display the structural variations.<br><br>Copy number variation and LOH: ONT reads (FASTQ format) from hetero-diploid strains were filtered and trimmed using NanoFilt software. LoxPsym-containing ONT reads were obtained by LAST software (v1250) for structural variant (SV) detection. To avoid alignment bias due to the similarities of ChrIII and SynIII, LAST was used to separate loxPsym-containing reads according to whether they contained SynIII PCRTags. Reads containing SynIII PCRTags were mapped to SynIII and were used to reconstruct SCRaMbLEd SynIII as previously described. Reads without SynIII PCRTags were aligned to the SparLox83R genome for SV calling using the NGMLR-sniffles (v1.0.12 for sniffles and v0.2.7 for ngmlr) pipeline. In addition, SynIII PCRTag-containing reads were aligned to the SparLox83R genome to detect translocation between SynIII and SparLox83R. LoxPsym sites that were not identified in any reads were considered to be the result of Loss of Heterozygosity (LOH). LoxPsym copy number variation (CNV) was identified using upstream and downstream 2000 bp flanking regions as described previously.<br><br>Hi-C data were mapped to the public S288C reference genome (GCF_000146045.2) using distiller-nf (v0.3.3).Read pairs that were not uniquely mapped (mapping score < 1) were discarded. Valid alignment files were transformed to .hic files for juicer using pre command. Hi-C map resolution was calculated as described previously59. Contact matrices used for heatmap visualization and further analysis were KR (Knight-Ruiz) normalized at 5 kb resolution. To compare the interaction frequencies between SparLox83R and other strains, the log2 ratio of a 5 kb binned contact map was computed and normalized by subtracting the median of its values.<br><br>For the construction of a 3D chromosome model, Hi-C data were mapped to the SparLox83R genome and binned at 5 kb resolution. Chromosomal 3D structures were then inferred using the Pastis (v0.4) method with a Poisson model and visualized using PyMOL.<br>CellProfiler v4.2.6 software was used to evaluate the intensities of colonies on plates.<br><br>For RNA seq, raw data adapters were removed using Cutadapter (v3.4) software. Cleaned data was mapped using Hisat2 (v2.2.1), and PCR duplicates were identified and eliminated using picard (v2.25.7). Gene read counts were generated by htseq-count software (v0.13.5) with the intersection-nonempty option, while log2(fold change) and padj values were calculated using DESEQ2 package (v1.30.1). TPM values for each gene were determined by Stringtie software (v2.1.7).

For manuscripts utilizing custom algorithms or software that are central to the research but not yet described in published literature, software must be made available to editors and reviewers. We strongly encourage code deposition in a community repository (e.g. GitHub). See the Nature Portfolio guidelines for submitting code & software for further information.

Data

Policy information about availability of data

All manuscripts must include a data availability statement. This statement should provide the following information, where applicable:

- Accession codes, unique identifiers, or web links for publicly available datasets
- A description of any restrictions on data availability
- For clinical datasets or third party data, please ensure that the statement adheres to our policy

The sequencing data that support the findings of this study have been deposited into NCBI BioSample database with the BioProject accession number: PRJNA768098 (https://www.ncbi.nlm.nih.gov/bioproject/PRJNA768098). The reference sequence of synIII used in this study can be downloaded from GenBank with accession codes KC880027 (https://www.ncbi.nlm.nih.gov/nuccore/KC880027). The complete dataset supporting the findings of this study can be found in both the main manuscript file and its Supplementary Information files. Source data are provided with this paper.

Research involving human participants, their data, or biological material

Policy information about studies with human participants or human data. See also policy information about sex, gender (identity/presentation), and sexual orientation and race, ethnicity and racism.

Reporting on sex and gender	N/A
Reporting on race, ethnicity, or other socially relevant groupings	N/A
Population characteristics	N/A
Recruitment	N/A
Ethics oversight	N/A

Note that full information on the approval of the study protocol must also be provided in the manuscript.

Field-specific reporting

Please select the one below that is the best fit for your research. If you are not sure, read the appropriate sections before making your selection.

☒ Life sciences ☐ Behavioural & social sciences ☐ Ecological, evolutionary & environmental sciences

For a reference copy of the document with all sections, see nature.com/documents/nr-reporting-summary-flat.pdf

Life sciences study design

All studies must disclose on these points even when the disclosure is negative.

Sample size	For the genomic stability analysis, 35 colonies were used for PCRtag analysis in Fig. 1C. We deem this number to be appropriate as convention in similar reports is n=30 (Science, 2014, PMID: 27013737).<br>For pooled SCRaMbLEd genome reconstruction and rearrangement analysis in Fig. 2, 774,791 (8.06 Gb) and 2,523,754 (22.5 Gb) reads were generated from the haploid and diploid SCRaMbLEant populations, respectively. 18,395 and 30,194 loxPsym-containing reads were detected in haploid and diploid strains, respectively. 2,850 potential rearrangement events may be took place in SparLox83. About 6.5 (18,395/2,850) and 10.6 times were detected for each potential rearrangement events in haploid and diploid strains, respectively.<br>For continuous evolution, n=48 represented 48 individual colonies from each strain.
Data exclusions	All data are included for analysis.
Replication	Experiments including serial dilution, Hi-C and RNA-seq were performed at least two times, as indicated in the figure legends and showed similar results.
Randomization	Nine clones with different colony size were picked at random from agar plates for whole genome sequencing in Fig. 4.
Blinding	Blinding is not relevant because no group allocation was involved in this study.

Reporting for specific materials, systems and methods

We require information from authors about some types of materials, experimental systems and methods used in many studies. Here, indicate whether each material, system or method listed is relevant to your study. If you are not sure if a list item applies to your research, read the appropriate section before selecting a response.

Materials & experimental systems

n/a	Involved in the study
☒	☐ Antibodies
☒	☐ Eukaryotic cell lines
☒	☐ Palaeontology and archaeology
☒	☐ Animals and other organisms
☒	☐ Clinical data
☒	☐ Dual use research of concern
☒	☐ Plants

Methods

n/a	Involved in the study
☒	☐ ChIP-seq
☒	☐ Flow cytometry
☒	☐ MRI-based neuroimaging

Plants

Seed stocks	*Report on the source of all seed stocks or other plant material used. If applicable, state the seed stock centre and catalogue number. If plant specimens were collected from the field, describe the collection location, date and sampling procedures.*
Novel plant genotypes	*Describe the methods by which all novel plant genotypes were produced. This includes those generated by transgenic approaches, gene editing, chemical/radiation-based mutagenesis and hybridization. For transgenic lines, describe the transformation method, the number of independent lines analyzed and the generation upon which experiments were performed. For gene-edited lines, describe the editor used, the endogenous sequence targeted for editing, the targeting guide RNA sequence (if applicable) and how the editor was applied.*
Authentication	*Describe any authentication procedures for each seed stock used or novel genotype generated. Describe any experiments used to assess the effect of a mutation and, where applicable, how potential secondary effects (e.g. second site T-DNA insertions, mosiacism, off-target gene editing) were examined.*
